# The effect of bitter almond (*Amygdalus communis* L. var. Amara) gum as a functional food on metabolic profile, inflammatory markers, and mental health in type 2 diabetes women: a blinded randomized controlled trial protocol

**DOI:** 10.1186/s13063-023-07085-7

**Published:** 2023-01-17

**Authors:** Saba Saati, Parvin Dehghan, Fatemeh Azizi-Soleiman, Majid Mobasseri

**Affiliations:** 1 Student Research Committee, Faculty of Nutrition and Food Science, Tabriz University of Medical Sciences, Tabriz, Iran; 2 Nutrition Research Center, Department of Biochemistry and Diet Therapy Faculty of Nutrition and Food Science, Tabriz University of Medical Sciences, Tabriz, 5166614711 Iran; 3 Department of Nutrition, School of Health, Arak University of Medical Sciences, Arak, Iran; 4 Department of Internal Medicine, School of Medicine, Tabriz University of Medical Sciences, Tabriz, Iran

**Keywords:** Type 2 diabetes, Inflammation, Oxidative stress, Bitter almond

## Abstract

**Background:**

Using functional foods in the prevention and treatment of type 2 diabetes mellitus (T2DM) has increased across the world owing to their availability, cultural acceptability, and lower side effects. The present study will aim to examine the impact of bitter almond (*Amygdalus communis* L. var. Amara) gum as a functional food on metabolic profile, inflammatory markers, and mental health in women with T2DM.

**Methods:**

We will conduct a randomized, triple-blind, placebo-controlled trial. A total of 44 women with T2DM will be randomly allocated into two groups: an intervention group (*n* = 20) and a placebo group (*n* = 20). Patients will receive either 5 g/d of bitter melon gum or a placebo for 8 weeks. Clinical and biochemical outcome parameters which include glycemic indices, lipid profile, inflammatory markers, oxidative stress indices, tryptophan (Trp), kynurenine (KYN), cortisol, glucagon-like peptide 1 (GLP-1), leptin, adiponectin, ghrelin, peroxisome proliferator-activated receptor (PPAR) gene expression, brain-derived neurotrophic factor (BDNF), endothelial cell adhesion molecules, plasminogen, cluster deference 4 (CD4), cluster deference 8 (CD8), anthropometric indices, blood pressure, dietary intake, and mental health will be measured at the baseline and end of the study. Statistical analysis will be conducted using the SPSS software (version 24), and *P* value less than 0.05 will be considered statistically significant.

**Discussion:**

The present randomized controlled trial will aim to investigate any beneficial effects of bitter almond gum supplementation on the cardio-metabolic, immune-inflammatory, and oxidative stress biomarkers, as well as mental health in women with T2DM.

**Ethics and dissemination:**

The study protocol was approved by the Ethical Committee of the Tabriz University of Medical Sciences (IR.TBZMED.REC.1399.726).

**Trial registration:**

Iranian Registry of Clinical Trials (www.irct.ir/IRCT20150205020965N7)

**Supplementary Information:**

The online version contains supplementary material available at 10.1186/s13063-023-07085-7.

## Background

Type 2 diabetes mellitus (T2DM), a metabolic disorder characterized by imbalanced blood sugar levels, is thought to be precipitated by a combination of impaired insulin secretion and insulin resistance [1]. Ethnicity, genetic predisposition, obesity, low physical activity, and unhealthy diet are involved in the pathogenesis of T2DM [2]. In 2019, it was estimated that 463 million people are living with T2DM worldwide [3]. This number has almost tripled over the last two decades and is projected to reach 578 million by 2030 and 700 million by 2045 [4]. The prevalence of T2DM has risen dramatically in countries experiencing epidemiologic transitions, including Asia, the Middle East, and North Africa [5]. The economic costs attributable to T2DM are also considerable. T2DM direct health care expenditure was estimated to be US$760 billion in 2019 [3]. Thus, developing novel adjuvant therapies for the management of T2DM and its associated complications is vital. Recent progress in understanding the development and progression of diabetes has shown a strong association between hyperglycemia, oxidative stress, inflammation, gut microbiota, and T2DM [6].

Intracellular hyperglycemia increases reactive oxygen species (ROS) production, promotes advanced glycation end-product formation and activation of protein kinase C, and enhances polyol pathway flux. ROS stimulates the generation of inflammatory mediators and adhesion molecules, oxidized low-density lipoprotein (LDL) formation, and insulin resistance [7]. As oxidative stress can induce an inflammatory process, inflammation can also induce oxidative stress [8]. Following activation of the immune system, innate immune system cells pro-inflammatory produce cytokines and chemokines, which stimulate the production of reactive oxygen and/or nitrogen species. Pro-inflammatory cytokines activate macrophages to eliminate pathogens via the generation of ROS [9]. Increased inflammatory markers may be related to mental health conditions [10]. Depressive disorders and impaired mental health-related quality of life are more prevalent among people with T2DM [11]. The relationship between T2DM and depression is thought to be bidirectional because of similar underlying pathological features in these conditions, including inflammation [12]. Higher serum levels and expression of inflammatory and pro-inflammatory markers in depressed patients with T2DM have been reported [13].

The microbiome may play an essential role in the development of T2DM [14]; an increase in the abundance of Firmicutes and Actinobacteria and a decrease in the proportion of Bacteroidetes can result in an inflammatory cascade, insulin resistance, and oxidative stress [15]. Potential mechanisms include modulation of inflammation, increased intestinal permeability, changed glucose metabolism, altered fatty acid oxidation, synthesis, and energy expenditure [16]. Gut dysbiosis results in an increase in the leakage of bacterial products such as lipopolysaccharides (LPS) into the bloodstream [17]. LPS is recognized by Toll-like receptor 4 (TLR4), which is implicated in inflammation [18]. It has been shown that LPS activate intracellular pathways of c-Jun N-terminal kinase (JNK) and IκB kinase (IKK)-β [19]. Activation of JNK promotes the serine phosphorylation of insulin receptor substrate (IRS)-1, which inhibits normal signal transduction through the insulin receptor/IRS-1 axis, which inhibits its action, and leads to insulin resistance [20]. Activation of the IKKβ pathway induces the activation of nuclear factor (NF)-κB and enhances the expression of numerous pro-inflammatory mediators that are involved in insulin resistance [21]. Therefore, modulation of the gut microbiota can be considered a potential therapeutic strategy. One way would be to improve the gut microbiome by using prebiotics or probiotics. Prebiotics are non-digestible and fermentable fibers and sugars that promote the growth and/or activity of a beneficial gut microbiome [22]. Prebiotics increase the growth of beneficial bacteria such as lactobacilli and bifidobacteria, prevent the proliferation of harmful bacteria, and allow beneficial bacteria to produce short-chain fatty acids (SCFAs), including acetate, butyrate, and propionate [23]. These SCFAs decrease inflammation, improve intestinal membrane integrity, and increase the absorption of nutrients. A systematic review showed that prebiotics might improve metabolic and inflammatory biomarkers in adult women with T2DM [24].

Nowadays, the use of herbal medicine is proposed to be an adjunct to conventional antidiabetic treatments [25]. Medicinal plants contain glycosides, alkaloids, terpenoids, flavonoids, and carotenoids, which can exert antidiabetic effects [26]. Bitter almond gum is a key herb in Iranian medicine which is extracted from *Amygdalus scoparia* Spach. Remarkable in vitro antioxidant, antimicrobial, and antitumor effects of the water, methanol, and ethanol extracts of bitter almond kernels were demonstrated by Gomaa et al. [27]. Bitter almond gum increased the survival rate of *Lactobacillus acidophilus* La5 in tomato juice mixtures during fermentation, refrigeration, and exposure to gastric juice [28]. Bitter almond gum is an arabinogalactan polysaccharide that has prebiotic properties [29]. Bitter almond gum may have a favorable immune modulator effect possibly related to its prebiotic content [29]. To the best of our knowledge, there is only one study that evaluated the effect of bitter almond gum on metabolic parameters in overweight hyperlipidemic patients. In this study, bitter almond gum-enriched juice consumption significantly reduced body weight, body mass index (BMI), serum triglyceride (TG), hyperinsulinemia, and homeostatic model assessment for insulin resistance (HOMA-IR). We hypothesized that bitter almond gum, as adjuvant therapy, is able to improve metabolic, inflammatory, and oxidative stress biomarkers, as well as mental health in T2DM women.

## Methods/design

### Ethical consideration

The trial protocol is approved by the Tabriz University of Medical Sciences Ethical Committee (IR.TBZMED.REC.1399.726). The clinical trial number is IRCT20150205020965N7, which has been registered on the Iranian Registry of Clinical Trials (IRCT) website (www.irct.ir/IRCT20150205020965N7). This study will be conducted in accordance with the Declaration of Helsinki. Written informed consent will be obtained from all participants prior to study entry and after patient education about the study objective, process, schedule, potential risks, and benefits.

### Protocol amendments

Any changes in the study protocol will be reviewed and approved by all authors. If any major change is made to the protocol, additional ethical approval must be obtained from the Ethics Committee.

### Dissemination

The results of this experience will be published in a peer-reviewed journal.

### Safety assessments

The dosage to be administered in this clinical trial is within the recommended range based on the previous studies [30]. Moreover, documentation of all potential adverse events reported by the patients will be recorded by investigators from baseline through the follow-up period. If the reported adverse events are associated with the use of bitter almond gum, patients will be asked to stop taking the supplement, and they will be referred to a physician for therapy. Subjects with adverse events will be followed until the events will be resolved. Serious adverse events will be followed up to determine the final outcome. In addition, any possible negative effects will be notified to the ethics committee.

### Study design and participants

**Fig. 1:**
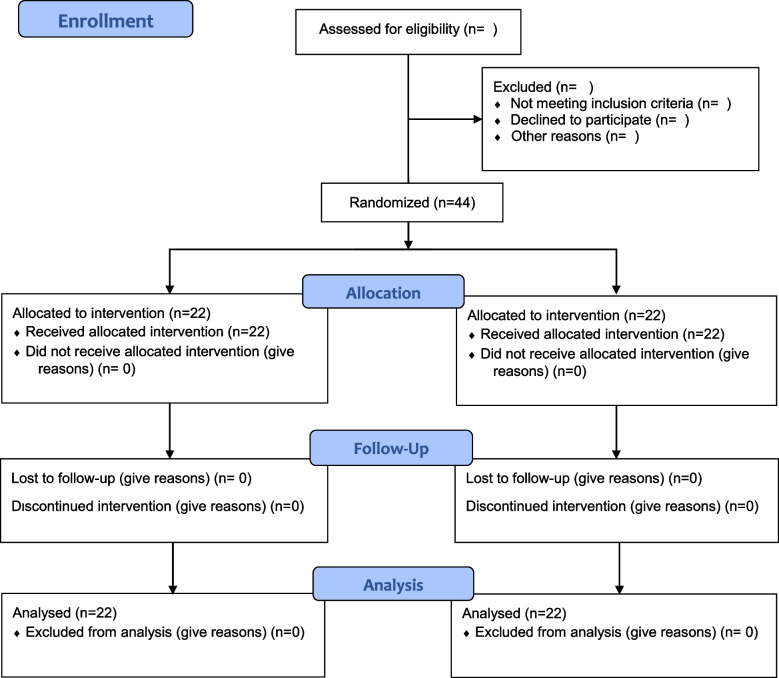
Consolidated Standards of Reporting Trials (CONSORT) diagram

The proposed study is a randomized, triple-blind, placebo-controlled, parallel-group clinical trial with a superiority framework to deliver an intention to treat (ITT) to evaluate the efficacy of bitter almond gum on cardio-metabolic, immune-inflammatory, and oxidative stress biomarkers, as well as mental health, compared with a placebo in women with T2DM. The allocation ratio will be 1:1. The study will begin later this year, 2022. Women with T2DM will be recruited from clinics of the Tabriz University of Medical Sciences, Iran, through public announcement systems and doctor referrals. A trained nutritionist will be served to include participants until the target sample size is reached and the project supervisor will assign participants to intervention groups based on the randomization list. The research design is presented in Fig. 1. This clinical trial protocol is based on the Standard Protocol Items: Recommendations for Interventional Trials (SPIRIT) 2013 checklist (Additional file 1: SPIRIT checklist) [31]. The diagram of the study protocol and initial and ongoing participant recruitment is demonstrated in Table 1.

**Table 1:**
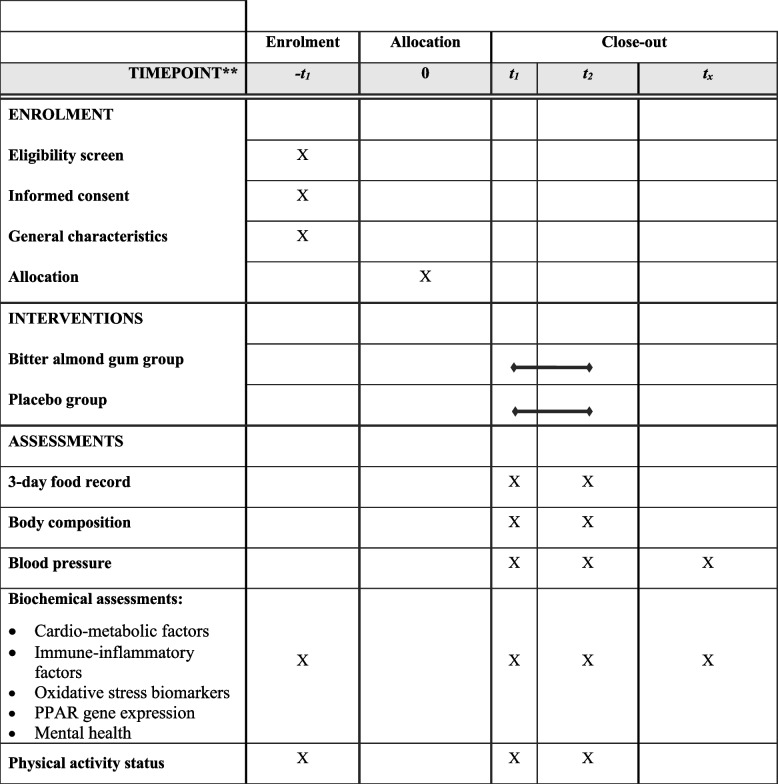
Standard Protocol Items: Recommendations for Interventional Trials (SPIRIT) chart for the study process. The “X” is indicating what is done in the given period

*PPAR* peroxisome proliferator-activated receptor

### Eligibility of criteria

Women aged between 30 and 65 years and diagnosed with T2DM (fasting blood sugar (FBG) ≥126 mg/dl [32]) that satisfy the inclusion criteria will be recruited. The inclusion criteria will be considered as follows: diagnosed with T2DM at least 6 months, body mass index (BMI) > 25 and < 35 kg/m^2^, no weight changes during the last three months, use of glucose-lowering drugs (including sulfonylureas, glinides, biguanides, thiazolidinediones, dipeptidyl-peptidase 4 inhibitors, sodium-glucose transporter 2 inhibitors, alpha-glucosidase inhibitors, amylin mimetics, and incretin mimetic), dietary fiber intake of < 25 g per day, and propensity to take bitter almond gum supplement during the study. Patients will be excluded if they use insulin, glucocorticoids, laxatives, anti-obesity drugs, nonsteroidal anti-inflammatory drugs (NSAIDs), antidepressants, and antibiotics; take supplements including multivitamins, n-3 fatty acids, Ginkgo biloba, antioxidants, probiotics, and other prebiotics in the previous 3 months before the recruitment; have a history of weight loss or dieting in the last 6 months; have cancers and gastrointestinal, thyroid, heart, kidney, liver, lung, and infectious diseases; consume alcohol and smoke; and are pregnant or lactating.

### Randomization and blinding

After obtaining the informed consent of the study participants, eligible subjects will complete a 2-week run-in period. During this time, patients will be requested to follow dietary and exercise advice according to recommendations for T2DM patients [33]. After the run-in period, the patients will be randomized to one of the two arms (bitter almond gum group or placebo group) in equal numbers, stratified by age and BMI. For stratified block randomization, random allocation software (RAS) will be used with block sizes of 2 and 4. Randomization will be performed by a third party that is not involved in this research. Block size will be concealed to all researchers until code-breaking. To enter a patient into the trial, one of the researchers will open the sequentially numbered sealed opaque envelopes. The study design is blinded to the main investigator, those who collect the data, the statistical consultant, and the participants.

### Intervention

Diabetic patients will receive either 5g/d (2.5 g at breakfast and 2.5 g at dinner) of bitter almond gum powder (bitter almond gum, Flavinea Co, Iran) or maltodextrin powder (placebo) (Jiujiang Hurirong Trade Co, China) for 2 months. Participants will be instructed to use supplements with semi-solid food like yogurt or salad. Both bitter almond gum and maltodextrin powders are odorless and tasteless and will be provided in identical opaque packages. Study participants will be informed on how to take their supplements. Patients will be followed by phone calls twice a week to ensure compliance with the intervention and dietary and physical activity guidelines and receive reports of possible adverse events experienced following bitter almond gum consumption. Compliance will be assessed based on a self-administered checklist and used packages by each person. Also, a checklist will be provided for participants to mark after each consumption of the prescribed supplement to evaluate for cases of non-compliance. Adherence to the regular consumption of powders (bitter almond gum or placebo) will also be determined by counting packages, also at least 10% of the supplements as noncompliant participants and, consequently, excluded from the study. Participants will be free to withdraw from participation in the study at any time upon request. Withdrawal criteria will be as follows: (1) Any clinical adverse event or medical condition occurs that the patient’s continued participation in the study would not be in the best interest of her and (2) the patient meets a newly developed or not previously recognized exclusion criterion that precludes further study participation. When the study ends, the results will be notified to all participants by post and published on a public website.

### Sample size

Given the absence of data in this field, we sought to determine the sample size of our study based on the study of Sheu et al. [34] selected because of its similarity to our intervention. We calculated the sample size by taking into account the LDL levels. Considering a power of 90%, a 95% confidence interval, and the changes in LDL values as one of the primary outcomes (mean difference of 28.3 mg/dl between the two groups), the required sample size was calculated to be 19 for each study group. To compensate for a drop-out rate of 15% throughout the study, we increased the final sample size to 22 patients in each group.

### Primary and secondary outcomes

Primary outcomes of the study will be FPG, glycosylated hemoglobin (HbA1c), and insulin. Secondary outcomes will include the lipid profile (total cholesterol (TC), high-density lipoproteins (HDL), low-density lipoproteins (LDL), TG), high-sensitivity C-reactive protein (hs-CRP), tumor necrosis factor-α (TNF-α), interleukin-1 (IL-1), interleukin-6 (IL-6), interleukin-10 (IL-10), interleukin-17 (IL-17), lipopolysaccharides (LPS), total antioxidant capacity (TAC), malondialdehyde (MDA), oxidative stress index (OSI), total oxidant status (TOS), superoxide dismutase (SOD), glutathione peroxidase (GSH-Px), catalase (CAT), soluble receptor for AGEs (sRAGE), carboxymethyl lysine (CML), pentosidine, 8-iso-prostaglandin F2α (8-iso-PGF2α), nitric oxide (NO), tryptophan (Trp), kynurenine (KYN), cortisol, glucagon-like peptide 1 (GLP-1), receptor for advanced glycation end products (RAGE), leptin, adiponectin, ghrelin, peroxisome proliferator-activated receptor (PPAR) gene expression, brain-derived neurotrophic factor (BDNF), endothelial cell adhesion molecules, plasminogen, cluster deference 4 (CD4), cluster deference 8 (CD8), anthropometric indices, blood pressure, dietary intake, and mental health. The following variables will be considered covariates: energy intake, weight changes, and the baseline values of glycemic indices, lipid profile, LPS, inflammatory and oxidative stress biomarkers, adipokines, and parameters related to mental health and the immune system. All variables will be measured at baseline and at the end of the study.

### Clinical, para-clinical assessment

Demographic details and current use of medications will be recorded at baseline. Evaluation of patients’ physical activity level (PAL) will be done at the onset and end of the study using the International Physical Activity Questionnaire Short-Form (IPAQ-SF) [35]. The Depression, Anxiety, and Stress Scale (DASS) and a general health questionnaire (GHQ) will be used for assessing patient mental health [36, 37]. The DASS questionnaire consists of 14 items that are divided into three subscales to measure depression, anxiety, and stress [38]. Additionally, four other subscales (somatic symptoms, anxiety, insomnia, social dysfunction, and severe depression) will be assessed using the 28-item GHQ [39]. A 3-day food diary (one for a weekend day and two for 2 non-consecutive weekdays) [40] will be completed by patients at baseline and at the end of the study and applied for dietary assessment. Collected dietary records will be analyzed using the “Nutritionist 4” software (First Databank Inc., Hearst Corp., San Bruno, CA, USA).

At the start and end of the study, weight and height will be measured to the nearest 0.1 kg and 0.1 cm employing a reliable digital scale (Seca, Hamburg, Germany) and a meter mounted on the wall, respectively. A non-elastic tape will be placed on the midpoint between the lowest rib and the upper iliac crest to measure the waist circumference (WC) [41]. The hip circumference (HC) will be recorded at the maximum circumference over the hips without pressing the skin. Neck circumference (NC) will be measured at the level of the mid-cervical spine below the cricoid cartilage, and a minimum circumference will be calculated to the nearest 0.1 cm [42]. The BMI will be derived from weight (kg) divided by the square of the height (m^2^). All the anthropometric variables will be measured by the same person to minimize measurement errors. Blood pressure will be measured three times by a DinaMap Compact after 30 min of resting, and the mean of the three will be reported.

A venous blood sample (10 mL) will be drawn from all participants following 10 to 12 h overnight fasting and centrifuged for serum isolation at baseline and at week 8 of the trial. FPG, lipid profile, and hs-CRP will be immediately determined after collecting samples. FPG and lipid profiles will be evaluated using the enzymatic colorimetric assay and commercial kits (Pars Azmoon, Tehran, Iran). The LDL will be derived from the Fried Ewald calculation [43]. The remainder of the serum samples will be stored at −70°C until the end of the study. Serum hs-CRP, TNF-α, IL-1, IL-6, IL-10, IL-17, LPS, CML, 8-iso-PGF2α, NO, Trp, KYN, cortisol, GLP-1, RAGE, leptin, adiponectin, ghrelin, BDNF, endothelial cell adhesion molecules, and plasminogen levels will be measured using an enzyme-linked immunosorbent assay (ELISA) kit. Flow cytometry and dual-color reagents will be applied to measure CD4 and CD8 (Dako Company, Denmark). HbA1c A will be determined using a high-pressure liquid chromatography D-10 system. Plasma insulin concentrations will be assessed using a chemiluminescent immunoassay method. TAC will be determined through the colorimetric method. Thiobarbituric acid reactive substances (TBARS) assay will be utilized to measure MDA levels using a spectrofluorometer [44]. The reverse transcription polymerase chain reaction (RT-PCR) will be used to determine the altered expression of the PPAR gene. The subsequent formulas will be used to calculate the oxidative stress index (OSI), the HOMA-IR, and the quantitative insulin sensitivity check index (QUICKI):

- OSI =100 × (TOS/TAC) [45]. Total oxidant status
- HOMA-IR = [fasting insulin (μU/mL) × FPG (mM/L)] / 22.5
- QUICKI = 1/ (log (fasting insulin, μU/ml) + log (FPG, mg/dl)) [46].

### Data management

The study progress, protocol, validity, and integrity of the info, also as ethical requirements, will occasionally be supervised by a clinical trial monitor. All participants will be encouraged to answer the questions honestly. To analyze the data of the participants who withdraw from this trial for any reason, the ITT analysis will be used. All participants will be followed up for 8 weeks after the treatment assignment.

### Confidentiality

Each participant will be assigned a unique code number. All data will be stored on the drive and available only to the study team. Questionnaire data and consent forms will be stored on paper, remain separate from study data, and be coded with a particular code number.

### Statistical analysis

SPSS version 24.0 (SPSS Inc., Chicago, IL, USA) will be applied to analyze the data. Mean ± standard deviation (SD) or median (25th–75th percentile) and frequency (percentage) will be used to present quantitative and qualitative variables, respectively. The normality of our data will be assessed with the Kolmogorov-Smirnov test. Log transformation in the cases of non-normally distributed data will be done. In order to examine differences in qualitative and quantitative baseline variables between groups, we will apply the chi-square test and the unpaired Student *T*-test, respectively. To compare quantitative variables between groups, an analysis of covariance (ANCOVA) will be performed post-intervention. For within-group comparison, we will conduct the paired sample Student’s *T*-test or its nonparametric equivalent, the Wilcoxon test. To find the percentage difference, the absolute value of the change will be divided by the average of the values and multiplied by 100 [100 × (intervention values − placebo values)/placebo values]. *P* value < 0.05 will be considered statistically significant.

### Patient and public involvement

Involving patients in designing, conducting, reporting, or disseminating will not be necessary for this study.

## Discussion

This study is a randomized controlled trial to evaluate the usefulness of bitter almond gum as a functional food on the cardio-metabolic, immune-inflammatory, and oxidative stress biomarkers, as well as mental health in women with T2DM. Documented studies suggest the beneficial effects of consuming functional food on macro- and micro-vascular complications of T2DM [47–49]. It is well established that prebiotics exerts wide-ranging impacts on human health and disease. There are numerous studies on the positive effects of prebiotics on cancer, vascular diseases, obesity, and mental disorders [50]. Bitter almond gum, as a possible source of polyphenols and prebiotic fiber, might have anticancer, anti-inflammatory, antioxidant, antilipidemic, antimicrobial, antiviral, and immunomodulatory properties [51, 52]. A randomized study found that 6-week bitter almond gum consumption significantly decreased anthropometric measures and insulin resistance in hyperlipidemic patients [30]. Flavonoids and phenolic acids activate the PPAR gene [53]. PPAR activates the expression of genes encoding lipoprotein lipase (LPL) and apo C-II oxidation [54]. These mechanisms might play a role in the improvement of the lipid profile after bitter almond gum supplementation. On the other hand, bitter almond gum, as a prebiotic, improves glucose tolerance via an increase in GLP-1 secretion [55]. Studies on the effect of bitter almond gum on health are rare. Prebiotic compounds detected in bitter almond gum resemble gum Arabic in being composed of the arabinogalactan polysaccharide [29]. Babiker et al. reported the glucose and lipid-lowering effect of Arabic gum in patients with T2DM [56]. Ahmed et al. revealed that Arabic gum supplementation could downregulate adipose triglyceride lipase (ATGL) and super conserved receptor expressed in brain2 (SREB2) and upregulate hormone-sensitive lipase (HSL) gene expression in the liver of mice fed a high-fat diet [57]. The positive effects of Arabic gum in reducing oxidative stress and inflammation have also been shown [58, 59]. An animal study evaluated the effect of Arabic gum on inflammatory and oxidative stress biomarkers in the gastrointestinal tract of the experimental model of chronic kidney disease (CKD), lower levels of TNF-α, IL-6, transforming growth factor β1 (TGF-β1), lipid peroxidation, nitrite, and higher concentrations of IL-10, catalase, glutathione reductase, TAC, SOD, and nuclear factor erythroid 2–related factor 2 reported in the duodenal mucosa [60]. Other anti-inflammatory effects attributed to Arabic gum include blockage of the liver macrophage function, modulation of nuclear factor-kB, the maturation of dendritic cells (DCs), and consequent improvement of CD4+ T cell proliferation [61]. Arabic gum may directly scavenge free radicals or ROSs through the presence of various antioxidant compounds or by elevating the synthesis of antioxidant biomolecules [62, 63]. It is possible that Arabic gum can improve the antioxidant capacity due to the presence of amino acid residues such as lysine, tyrosine, and histidine, which show antioxidant properties [64, 65]. This trial is the first randomized, triple-blind controlled study investigating the effects of bitter almond gum consumption on the cardio-metabolic risk factors, oxidative stress, inflammatory biomarkers, LPS, CML, 8-iso-PGF2α, NO, Trp, KYN, cortisol, GLP-1, RAGE, leptin, adiponectin, ghrelin, BDNF, and mental health in women with T2DM. We hypothesize that bitter almond gum supplementation would improve cardio-metabolic, inflammatory, and oxidative stress indices and mental health through gut microbiota modulation in T2DM women.

## Strengths and limitations of the study

The strengths of our trial include using a triple-blind, placebo-controlled, randomized clinical design and also exploring the effects of bitter almond gum supplementation on metabolic parameters, inflammatory markers, and mental health in women with T2DM for the first time. Also, as there is a need to find promising therapeutic agents for managing diabetes and its complications, the application of bitter almond gum might be a natural product to achieve this goal. However, there are some limitations to this research. First, self-reported dietary and physical activity habits may impact the results. Second, there is no compliance biomarker for measuring bitter almond gum intake. Finally, a longer duration of bitter almond gum supplementation might be advisable to find changes in trial biomarkers.

## Conclusion

We hope bitter almond gum supplementation will improve metabolic parameters, inflammation, oxidative stress, and mental health in women with T2DM. We expect that the results of this trial will provide scientific evidence in support of bitter almond gum intake for the management of T2DM and its comorbidities.

## Trial status

The present protocol is version 1, dated Aug 25, 2022. Recruiting of participants is underway, but the trial has not yet started.

## Supplementary Information

**Additional file 1.** SPIRIT checklist.

## Data Availability

Related articles will be published with all data generated or analyzed during this study.

## Abbreviations

8-iso-PGF2α: 8-Iso-prostaglandin F2α
ATGL: Adipose triglyceride lipase
ANCOVA: Analysis of covariance
BMI: Body mass index
BDNF: Brain-derived neurotrophic factor
CML: Carboxymethyl lysine
CKD: Chronic kidney disease
CD4: Cluster deference 4
CD8: Cluster deference 8
GLP-1: Glucagon-like peptide 1
DCs: Dendritic cells
DASS: Depression, Anxiety, and Stress Scale
ELISA: Enzyme-linked immunosorbent assay
FPG: Fasting plasma glucose
GHQ: General health questionnaire
HbA1c: Glycosylated hemoglobin
HDL: High-density lipoproteins
hs-CRP: High-sensitivity C-reactive protein
HC: Hip circumference
HOMA-IR: Homeostasis model assessment insulin resistance
HSL: Hormone-sensitive lipase
IKK: IκB kinase
IRS: Insulin receptor substrate
ITT: Intention to treat
IL-1: Interleukin-1
IL-10: Interleukin-10
IL-17: Interleukin-17
IL-6: Interleukin-6
IDF: International Diabetes Federation
IPAQ-SF: International Physical Activity Questionnaire Short-Form
KYN: Kynurenine
LPS: Lipopolysaccharides
LPL: Lipoprotein lipase
LDL: Low-density lipoprotein
MDA: Malondialdehyde
NC: Neck circumference
NF: Nuclear factor
NO: Nitric oxide
PPAR: Peroxisome proliferator-activated receptor
PAL: Physical activity level
ROS: Reactive oxygen species
RT-PCR: Reverse transcription polymerase chain reaction
AGE: Receptor for advanced glycation end products
SPIRIT: Standard Protocol Items: Recommendations for Interventional Trials
TG: Triglyceride
TLR4: Toll-like receptor 4
SCFAs: Short-chain fatty acids
SD: Standard deviation
SREB2: Super conserved receptor expressed in brain2
TBARS: Thiobarbituric acid reactive substances
TAC: Total antioxidant capacity
TC: Total cholesterol
TGF-β1: Transforming growth factor β1
Trp: Tryptophan
TNF-α: Tumor necrosis factor-α
T2DM: Type 2 diabetes mellitus
WC: Waist circumference
